# CRISP: A checklist for primary health care and family medicine research in Africa and worldwide

**DOI:** 10.4102/phcfm.v16i1.4790

**Published:** 2024-11-21

**Authors:** William R. Phillips, Elizabeth Sturgiss

**Affiliations:** 1 Department of Family Medicine, University of Washington, Seattle, United States; 2 School of Primary and Allied Health Care, Monash University, Clayton, Australia

Primary health care (PHC) is a universal paradigm that meets universal human needs. Family medicine (FM) is a distinct clinical speciality committed to continuous, comprehensive care that is patient-centred, community-oriented and evidence-based. Both require foundations built on primary care (PC) research.

Primary care research – like PC and PHC systems – lacks the recognition and support that reward research focused on diseases, organs or specialities. Primary care research must survive with fewer dedicated institutes or national programmes, less funding and fewer training opportunities. Support is limited for practice-based and clinician-led research. These disadvantages impede progress across the global PC research community, particularly in Africa.

Despite such disadvantages, PC research is growing across Africa^1^ and has the potential to conduct even more interdisciplinary, international and community-engaged research. Needs and opportunities are great for research on the fundamentals of FM, including continuity and coordination of whole-person care, and on the principles of PHC, including community engagement, multisectoral collaboration and whole-population care.^2^ In addition to informing clinical care, PC research is needed to advance organisational leadership and management.

For research to improve patient care and health outcomes, the findings must be reported and disseminated for implementation in clinical practice and healthcare systems. International studies show that family physicians rely on reports of original research but often find that published reports fail to include the information necessary for application to PC.^3^ Furthermore, poor reporting contributes to research waste, a problem recognised across biomedical sciences^4^ and critical in resource-poor settings. Study reports must be complete and transparent to ensure the findings are useful to readers. Primary care research reports must be applicable across the broad variety of clinical problems, patient populations and settings of PHC.

The international, interdisciplinary movement to develop research reporting guidelines helps address these widely recognised needs. The EQUATOR Network hosts an online repository of checklists, most focused on specific study designs, health issues or disciplines (https://www.equator-network.org).

The CRISP (Consensus Reporting Items for Studies in Primary Care) checklist^5^ was developed by and for the international PC community (https://crisp-pc.org). The CRISP Working Group developed the checklist through 5 years of collaboration and consensus building that engaged over 300 participants from 29 nations in multiple studies.

The CRISP checklist contains 12 items identified by consensus as essential for reports of PC research. The list is flexible and adapts to the great variety of PC research methods, study designs, clinical problems and populations. Not every item will apply to every study. Consensus Reporting Items for Studies in Primary Care respects the breadth and depth of PC research and leaves the final content and format to the discretion of authors and editors. To meet the needs of PHC, the CRISP checklist covers items not included in other reporting guidelines. Therapeutic relationships, PC teams and the involvement of patients and communities in research are examples of items important to PC and unique to CRISP. The checklist can be used in conjunction with method-specific guides to ensure the research report contains key information useful to PC clinicians, researchers and policymakers.

A central theme across our CRISP studies emphasised that the description of context is essential for understanding the meaning of PC research. Many colleagues outside of the USA expressed frustration that reports of PC research did not include enough description of context to allow them to translate the findings to their own settings. Consensus Reporting Items for Studies in Primary Care emphasises description of the context of the setting and system in which the research took place. Context, always important in PHC, can be even more critical in many settings in Africa.

The *African Journal of Primary Health Care & Family Medicine* supports this initiative and encourages authors to consider the CRISP checklist as a tool to ensure that research reports contain relevant and valuable information for readers and users of PC research.

The CRISP checklist can also be a valuable tool for research training and study design. It provides one more tool to help meet the challenges facing PC research across Africa and around the world.

Primary health care is an evolving enterprise, and the CRISP checklist is a living document. We value the recognition of CRISP by the editorial team at the *African Journal of Primary Health Care & Family Medicine.* The CRISP Working Group welcomes feedback on the usefulness and applicability of the checklist items in the African setting.

The CRISP checklist can provide a structure to help design, conduct and report PC studies. It can help advance international, interdisciplinary, community-engaged research in areas of need for researchers, clinicians and leaders working in Africa. We hope it can help empower PHC researchers worldwide to meet the needs of clinicians, systems, patients and communities we serve.
